# Supplementary material for: Hybrid Models and Biological Model Reduction with PyDSTool
Source: PLoS Comput Biol. 2012 Aug 9;8(8):e1002628. doi: 10.1371/journal.pcbi.1002628 (PMC3415397; doi:10.1371/journal.pcbi.1002628)
Supplement: Text S4 — Complete source code for the PyDSTool package (version 0.88.120504). Includes API documentation and help files linking to web pages. This file is identical to the current public release on Sourceforge.net. (ZIP) [file pcbi.1002628.s004.zip › PyDSTool/html/PyDSTool.Toolbox.FSM.ExceptionFSM-class.html]

xml version="1.0" encoding="ascii"?


PyDSTool.Toolbox.FSM.ExceptionFSM


| Home | Trees | Indices | Help | PyDSTool |
| --- | --- | --- | --- | --- |

Package PyDSTool ::
Package Toolbox ::
Module FSM ::
Class ExceptionFSM

[hide private]

[frames] | no frames]

# Class ExceptionFSM

source code

```
              object --+        
                       |        
exceptions.BaseException --+    
                           |    
        exceptions.Exception --+
                               |
                              ExceptionFSM
```

---

This is the FSM Exception class.


|  |  |
| --- | --- |
| Instance Methods | [hide private] |

|  |  |
| --- | --- |
| \_\_init\_\_(self, value)  x.\_\_init\_\_(...) initializes x; see x.\_\_class\_\_.\_\_doc\_\_ for signature | source code |

|  |  |
| --- | --- |
| \_\_str\_\_(self)  str(x) | source code |

**Inherited from `exceptions.Exception`**:
`__new__`

**Inherited from `exceptions.BaseException`**:
`__delattr__`,
`__getattribute__`,
`__getitem__`,
`__getslice__`,
`__reduce__`,
`__repr__`,
`__setattr__`,
`__setstate__`

**Inherited from `object`**:
`__hash__`,
`__reduce_ex__`


|  |  |
| --- | --- |
| Properties | [hide private] |

**Inherited from `exceptions.BaseException`**:
`args`,
`message`

**Inherited from `object`**:
`__class__`


|  |  |
| --- | --- |
| Method Details | [hide private] |

|  |  |
| --- | --- |
| \_\_init\_\_(self, value)  *(Constructor)* | source code |

x.\_\_init\_\_(...) initializes x; see x.\_\_class\_\_.\_\_doc\_\_ for
signature

Overrides: object.\_\_init\_\_: *(inherited documentation)*

|  |  |
| --- | --- |
| \_\_str\_\_(self)  *(Informal representation operator)* | source code |

str(x)

Overrides: object.\_\_str\_\_: *(inherited documentation)*

  


| Home | Trees | Indices | Help | PyDSTool |
| --- | --- | --- | --- | --- |

|  |  |
| --- | --- |
| Generated by Epydoc 3.0.1 on Fri May 4 15:24:08 2012 | http://epydoc.sourceforge.net |
